# Infants with biliary atresia exhibit an altered amino acid profile in their newborn screening

**DOI:** 10.1007/s11306-024-02175-2

**Published:** 2024-10-05

**Authors:** Marie Uecker, Cornelia Prehn, Nils Janzen, Jerzy Adamski, Gertrud Vieten, Claus Petersen, Joachim F. Kuebler, Omid Madadi-Sanjani, Christian Klemann

**Affiliations:** 1 Department of Pediatric Surgery, Hannover Medical School, Carl-Neuberg-Str. 1, 30625 Hannover, Germany; 2 Metabolomics and Proteomics Core, Helmholtz Zentrum München, German Research Center for Environmental Health, Neuherberg, Germany; 3 Screening-Labor Hanover, Hanover, Germany; 4 Department of Clinical Chemistry, Hanover Medical School, Hanover, Germany; 5 Division of Laboratory Medicine, Centre for Children and Adolescents, Kinder- und Jugendkrankenhaus Auf der Bult, Hanover, Germany; 6 Institute of Experimental Genetics, Helmholtz Zentrum München, German Research Center for Environmental Health, Ingolstädter Landstraße 1, 85764 Neuherberg, Germany; 7 Department of Biochemistry, Yong Loo Lin School of Medicine, National University of Singapore, 8 Medical Drive, Singapore, 117597 Singapore; 8 Institute of Biochemistry, Faculty of Medicine, University of Ljubljana, Vrazov trg 2, 1000 Ljubljana, Slovenia; 9 Clinic for Paediatric Surgery and Paediatric Urology, Klinikum Bremen-Mitte, Bremen, Germany; 10 Department of Pediatric Surgery, University Medical Center Hamburg-Eppendorf, Hamburg, Germany; 11 Department for Pediatric Immunology, -Rheumatology & -Infectiology, Hospital for Children and Adolescents, Leipzig University, Leipzig, Germany; 12 Department of Human Genetics, Hannover Medical University, Hannover, Germany

**Keywords:** Dried blood spot analysis, Pediatric hepatology, Hypermethionemia, Targeted metabolomics

## Abstract

**Introduction:**

Biliary atresia (BA) is a rare progressive neonatal cholangiopathy with unknown pathophysiology and time of onset. Newborn Screening (NBS) in Germany is routinely performed in the first days of life to identify rare congenital diseases utilizing dried blood spot (DBS) card analyses. Infants with biliary atresia (BA) are known to have altered amino acid profiles (AAP) at the time point of diagnosis, but it is unclear whether these alterations are present at the time point of NBS.

**Objectives:**

We aimed to analyze amino acid profiles in NBS–DBS of infants with Biliary Atresia.

**Methods:**

Original NBS-DBS cards of 41 infants who were later on diagnosed with BA were retrospectively obtained. NBS–DBS cards from healthy newborns (*n* = 40) served as controls. In some BA infants (*n* = 14) a second DBS card was obtained at time of Kasai surgery. AAP in DBS cards were analyzed by targeted metabolomics.

**Results:**

DBS metabolomics in the NBS of at that time point seemingly healthy infants later diagnosed with BA revealed significantly higher levels of Methionine (14.6 ± 8.6 μmol/l), Histidine (23.5 ± 50.3 μmol/l), Threonine (123.9 ± 72.8 μmol/l) and Arginine (14.1 ± 11.8 μmol/l) compared to healthy controls (Met: 8.1 ± 2.6 μmol/l, His: 18.6 ± 10.1 μmol/l, Thr: 98.1 ± 34.3 μmol/l, Arg: 9.3 ± 6.6 μmol/l). Methionine, Arginine and Histidine showed a further increase at time point of Kasai procedure. No correlation between amino acid levels and clinical course was observed.

**Conclusion:**

Our data demonstrate that BA patients exhibit an altered AAP within 72 h after birth, long before the infants become symptomatic. This supports the theory of a prenatal onset of the disease and, thus, the possibility of developing a sensitive and specific NBS. Methionine might be particularly relevant due to its involvement in glutathione metabolism. Further investigation of AAP in BA may help in understanding the underlying pathophysiology.

**Supplementary Information:**

The online version contains supplementary material available at 10.1007/s11306-024-02175-2.

## Introduction

Biliary atresia (BA) is a progressive liver disease in infants caused by obstruction of the extrahepatic bile ducts. Therapy in the neonatal period consists of the Kasai portoenterostomy which aims at establishing bile flow and prolonging survival with the patients’ own liver. However, most cases ultimately require a liver transplantation due to progressive liver damage, rendering BA the most common indication for pediatric liver transplantation (Petersen & Davenport, 2013).

Despite extensive research efforts, causes and pathogenesis of the disease remain elusive. Timely referral to a specialized center and performance of early Kasai procedure (< 60 days of life) is one of the few established factors considered relevant for the long-term outcome (Kelley-Quon et al., 2022; Lampela et al., 2012). Early diagnosis is therefore essential in order to provide timely surgery. The most important early symptoms of BA are jaundice and pale stools, both of which tend to develop gradually and are initially often unnoticed or misinterpreted for physiological newborn icterus during the neonatal period. Combined with a low incidence of the disease of about 1 in 20,000 live births (Jimenez-Rivera et al., 2013), diagnosis and referral of patients to specialized treatment centers are often delayed (Madadi-Sanjani et al., 2019; Serinet et al., 2009).

Since clinical symptoms only seem to develop during the first weeks of life, the theory of BA pathogenesis is commonly based on a yet unidentified trigger striking postnatally, such as a virus infection or toxins which then induce an abnormal inflammatory response in the bile ducts and liver leading to BA (Asai et al., 2015). Some authors differentiate between these “aquired” forms of BA and “congenital” BA which has a prenatal onset and is thought to affect around 10% of BA patients (Asai et al., 2015). However, in a study analyzing over 50% of a BA cohort for bilirubin levels during the first few days of life, all of the subjects exhibited elevated levels of direct bilirubin in serum, suggesting a prenatal onset of the disease in a larger proportion of patients (Harpavat et al., 2011). Case reports of abnormalities of the biliary system on fetal ultrasound (Morel et al., 2015) or abnormal gamma-glutamyl transferase in the amniotic fluid of infants diagnosed with BA postnatally (Muller et al., 1991) further support the theory of prenatal onset, underlining the need for an early screening parameter despite lacking consensus on the timing of BA onset.

Newborns in Germany are routinely screened for rare metabolic diseases using dried blood spot (DBS) cards within 36–72 h after birth (Spiekerkötter & Krude, 2022). BA is not part of the standardized newborn screening (NBS) as appropriate metabolites specific enough to detect affected patients directly after birth have not been identified yet. However, since early diagnosis is one of the most important prognostic factors, it is clearly desirable to incorporate BA into the screening process.

The research field of metabolomics has substantially advanced the study of specific metabolic pathways and cellular processes by generating highly complex data through analysis of tiny changes in low molecular weight metabolites (Hollywood et al., 2006). Metabolomics have provided new insights into a range of medical fields, such as oncology, tuberculosis research, or forensic medicine (Kumar & Misra, 2019; Preez et al., 2017; Szeremeta et al., 2021).

Amino acids are a subgroup of metabolomic analysis. Liver damage is known to cause amino acid imbalances due to various factors, such as changes in entero-hepatic circulation or loss of hepatocyte function with impaired hepatic amino acid metabolism (Holecek, 2015). Significant differences in amino acid profiles have been demonstrated for BA patients compared with healthy controls (Byrd et al., 1993; Weisdorf et al., 1987; D. Zhao et al., 2014). However, the youngest cohort studied were infants with a mean age of about 40 days. No data exists regarding amino acid profiles in BA patients during the immediate newborn period.

Combining the search for an improved screening option with exploring potential early amino acid level changes in BA we used metabolomic analysis of DBS cards collected within 72 h after birth to examine amino acid profiles in healthy newborns and infants later diagnosed with BA.

## Methods

The study protocol is in accordance with the declaration of Helsinki and was approved by the institutional ethics committee (No.41/2000). All parents’ or legal guardians’ written consent was obtained.

The study was conducted over a period of 5 years (March 2016–February 2021). When infants presented at our institution with a diagnosis of BA for the Kasai procedure, DBS cards from the NBS were retrospectively acquired from the respective screening laboratories at their place of birth. Additionally, DBS samples were taken from BA patients on a fresh NBS card at the time of surgery. For the comparative cohort, anonymized sex-matched NBS cards from healthy infants were collected via the screening laboratory Hannover. All screening cards were cataloged and stored at − 80 °C after arrival at our laboratory. The time of previous exposure to room temperature was noted and later correlated to the results. DBS cards obtained at time of Kasai surgery were allowed to air dry for at least 2 h at room temperature protected from direct sunlight and subsequently stored at − 80 °C.

Data analysis for BA patients included sex, time of diagnosis, age at surgery, ISHAK-Score, as well as long-term outcome.

### Metabolomics measurements

Targeted metabolomics measurements were performed using liquid chromatography- and flow injection-electrospray ionization-tandem mass spectrometry (LC- and FIA-ESI-MS/MS) and the AbsoluteIDQ™ p180 Kit (BIOCRATES Life Sciences AG, Innsbruck, Austria). For the LC-part, compounds were identified and quantified based on scheduled multiple reaction monitoring measurements (sMRM), for the FIA-part on MRM. The complete assay procedures as well as the DBS extraction, have been previously published (Zukunft et al., 2013). In brief, freshly punched DBS disks (3 mm diameter) were placed into the cavities of the 96-well filter plate of the p180 assay. Amino acids in the samples were derivatized with an excess of 5% phenylisothiocyanate for 20 min and dried under a nitrogen stream. Samples were extracted for 30 min at RT with 300 µL methanol containing 5 mM ammonium acetate. The LC run was performed using an Agilent XDB-C18 column (3 × 100 mm, 3.5 µm). Sample handling was performed by a Hamilton Microlab STAR™ robot (Hamilton Bonaduz AG, Bonaduz, Switzerland) and an Ultravap nitrogen evaporator (Porvair Sciences, Leatherhead, U.K.), besides standard laboratory equipment. Mass spectrometric analyses were done on an API 4000 triple quadrupole system (SCIEX Deutschland GmbH, Darmstadt, Germany) equipped with a 1260 Series HPLC (Agilent Technologies Deutschland GmbH, Böblingen, Germany) and a HTC-xc PAL autosampler (CTC Analytics, Zwingen, Switzerland) controlled by the software Analyst 1.6.2. Data evaluation for quantification of metabolite concentrations and quality assessment were performed with the software MultiQuant 3.0.1 (SCIEX) and the MetIDQ™ software package, which is an integral part of the AbsoluteIDQ™ Kit. Metabolite concentrations were calculated using internal standards and reported in µmol/L (µM). The concentration values for DBS refer to the concentrations in the original full blood samples, which have been calculated from the three μL blood that contained the spot used for measurements.

### Statistics

The results were analyzed using GraphPad Prism software Version 9.0 (GraphPad Software, San Diego, CA) for the calculation of correlation coefficients for clinical data and comparison between healthy and BA groups using unpaired and paired* t*-test. Results are reported as means and standard deviations (SD), *p* < 0.05 was considered statistically significant.

## Results

In total, 95 DBS cards were collected, consisting of 41 NBS–DBS cards from infants diagnosed with BA, 14 DBS cards from BA patients collected at the time of Kasai Portoenterostomy (KPE), and 40 NBS–DBS cards from healthy newborns.

### Clinical data

**Fig. 1:**
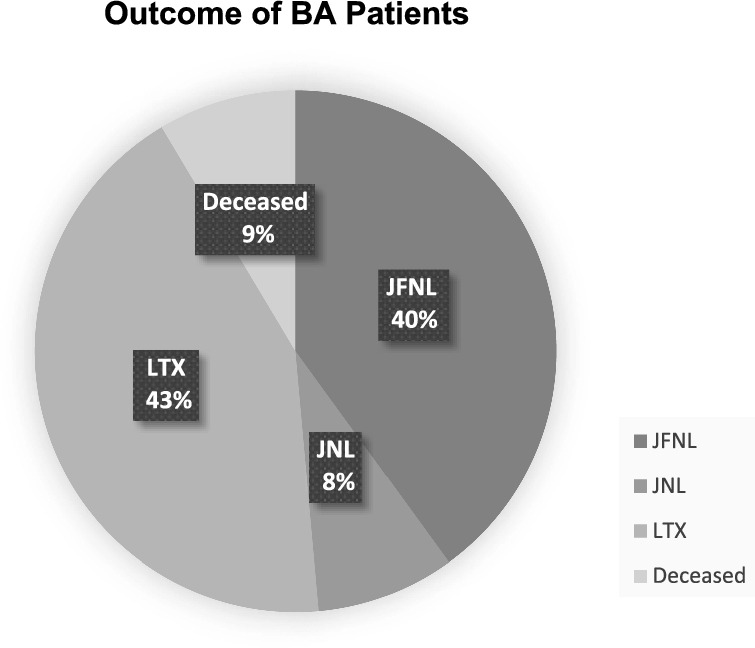
Clinical outcome of BA patients with acquired NBS card during a mean follow-up period of 45 (± 20) months (*n* = 35; JFNL = jaundice free native liver survival, JNL = jaundice native liver survival, LTX = Liver transplantation)

Clinical characteristics of BA patients are listed in Table 1. Mean age at surgery for BA patients was 57 (± 18) days. Long-term follow-up was available for 85% (*n* = 35) of patients; the mean follow-up period was 45 (± 20) months. Three patients (7%) died after surgery due to complications of comorbidities (congenital heart disease, *n* = 2) or terminal liver failure (*n* = 1) at the age of 3 and 5 months, and 20 months respectively. Fifteen patients (36%) were transplanted during the follow-up period at a mean of 580 days (± 602 days) after the Kasai procedure. Almost half of the patients (48%, *n* = 17) at follow-up were alive with their native liver, of which 15 patients (40%) were jaundice-free (Bilirubin < 20 μmol/l) (Fig. 1).

**Table 1:** Demographic table for BA patients with acquired NBS card from after birth (*n* = 41)

	*n* = 41
Gender	
Male	34% (*n* = 14)
Female	66% (*n* = 27)
Age at KPE (mean, in days)	55 ± 18
Bilirubin levels at KPE (mean, in μmol/l)	152 ± 43
ISHAK-score at KPE (range 1–6)	3.7 (± 1.4)
Cystic form of BA	7% (*n* = 3)
Syndromal form of BA	17% (*n* = 7)

*KPE*  Kasai Portoenterostomy

### Stability of analytes in NBS

**Fig. 2:**
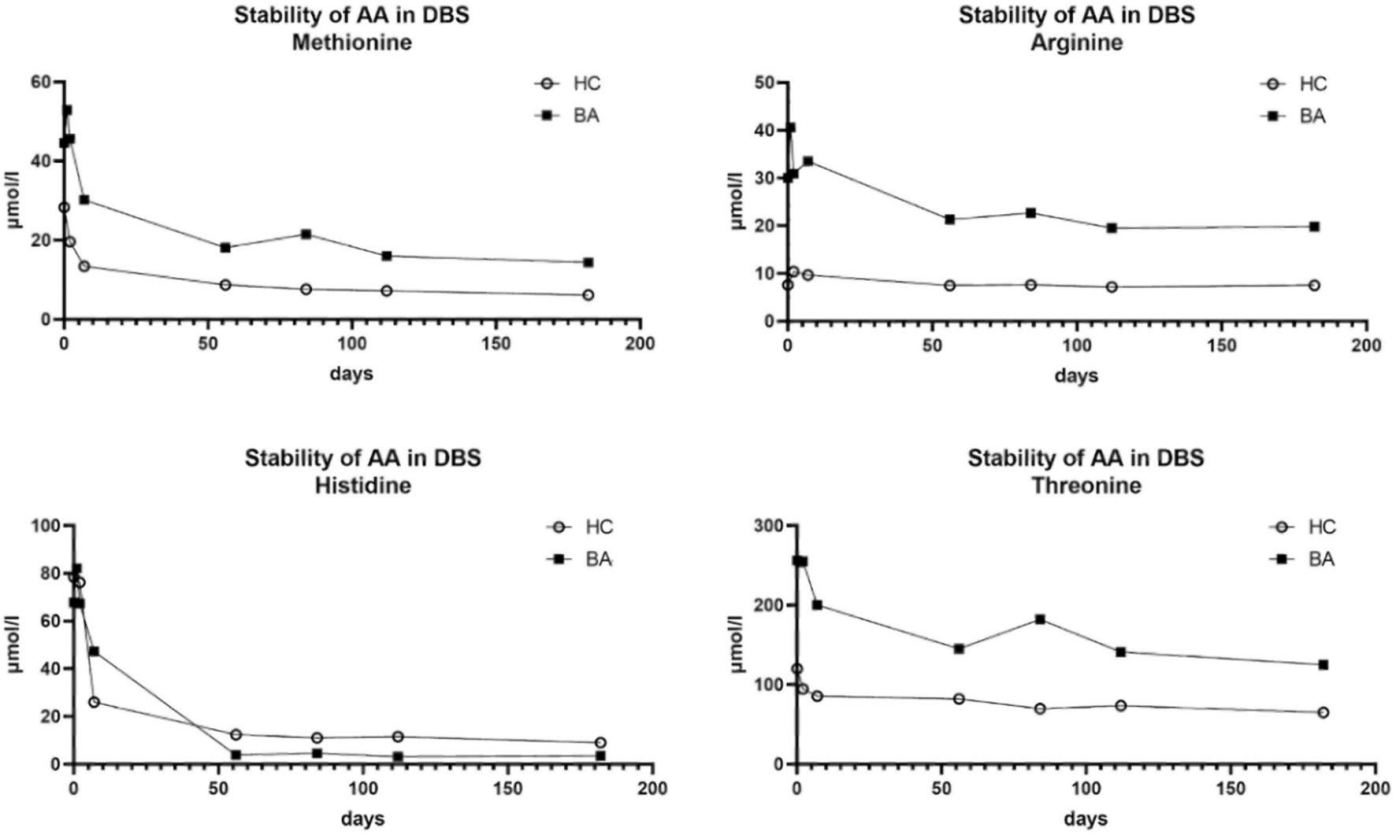
Stability of amino acid levels in NBS–DBS cards during exposure to room temperature over time. (AA: amino acids, DBS: dried blood spots, HC: healthy controls, BA: Biliary Atresia)

After completion of NBS analysis DBS cards are stored at room temperature in the screening centers. NBS cards of patients with BA were retrieved and stored at − 80 °C at our laboratory after a mean of 116 days (± 90 days). Therefore, we first assessed the stability of the analytes in screening cards of healthy and diseased children over time using dried blood spots from a BA patient at time of Kasai surgery and a healthy age-matched control taken during routine surgery (inguinal hernia repair). Individual blood spots of these patients were stored at − 80 °C after different time intervals (0 h, 2 h, 24 h, 48 h, up to 6 months). Results demonstrated a relevant decrease in the concentration of targeted metabolites during exposure to room temperature during the first days of storage. After approximately 50 days of storage, i.e. the storage interval of the cards analyzed in our experiment (116 days ± 90 days), levels were almost constant (exemplary data in Fig. 2, rest of data not shown).

### Increased levels of methionine, histidine, arginine, and threonine in the DBS–NBS in infants with BA

**Fig. 3:**
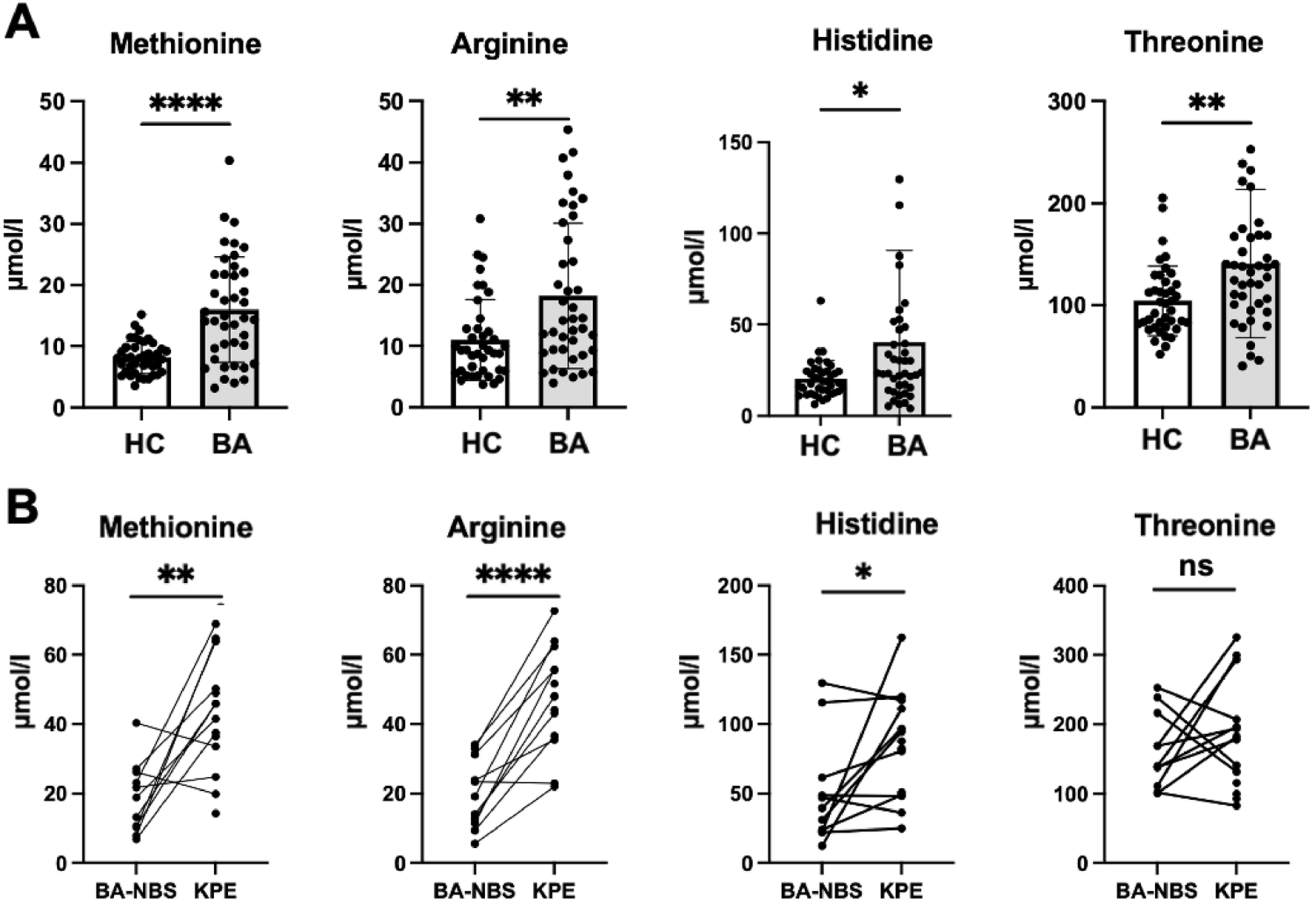
**a** The levels of Methionine, Histidine, Arginine, and Threonine in the NBS DBS in infants with BA are increased in newborns later clinically diagnosed with BA (*n* = 41) compared to healthy controls (*n* = 40). (unpaired *t*-test; ****p < 0.0001, **p < 0.01, *p < 0.05). **b** Changes in levels of the four significantly altered amino acids from birth to the time point of KPE. Each connected value belongs to the same BA patient at birth and at KPE. (*n* = 14, mean age at KPE 57 ± 18 days). (paired *t*-test; ****p < 0.0001, **p < 0.01, *p < 0.05, ns = not significant). (HC: healthy controls, BA: Biliary Atresia, BA-NBS: Biliary Atresia Newborn Screening, KPE: Kasai Portoenterostomy)

Retrospective analysis of the DBS–NBS by targeted metabolomics revealed significantly higher concentrations of Methionine, Arginine, Histidine and Threonine in infants with BA (Met: 14.6 ± 8.6 μmol/l, Arg: 14.1 ± 11.8 μmol/l, His: 23.5 ± 50.4 μmol/l, Thr: 132.9 ± 72.8 μmol/l) compared to healthy newborns (Met: 8.1 ± 2.6 μmol/l, Arg: 9.3 ± 6.6 μmol/l, His: 18.6 ± 10.1 μmol/l, Thr: 98.1 ± 34.3 μmol/l) (Fig. 3a). In BA infants, Methionine, Arginine and Histidine showed a significant further increase at time of KPE (Met: 42.6 ± 16.5 μmol/l, Arg: 47.3 ± 14.7 μmol/l, His: 83.0 ± 38.1 μmol/l) compared to time of newborn screening (Met: 18.8 ± 10.2 μmol/l, Arg: 19.9 ± 10.01 μmol/l, His: 50.4 ± 38.6 μmol/l), while changes in Threonine levels remained insignificant (Fig. 3b). The latter results are to be interpreted with caution as values at birth were measured in DBS cards stored at room temperature for longer periods than the DBS cards taken at KPE. At this point in time the methodical weakness of different room temperature storage time of the cards cannot be eliminated. However, since our stability tests showed a relevant decrease of metabolites during longer storage at room temperature, differences between the two groups may actually be more significant than the measured data suggests.

**Fig. 4:**
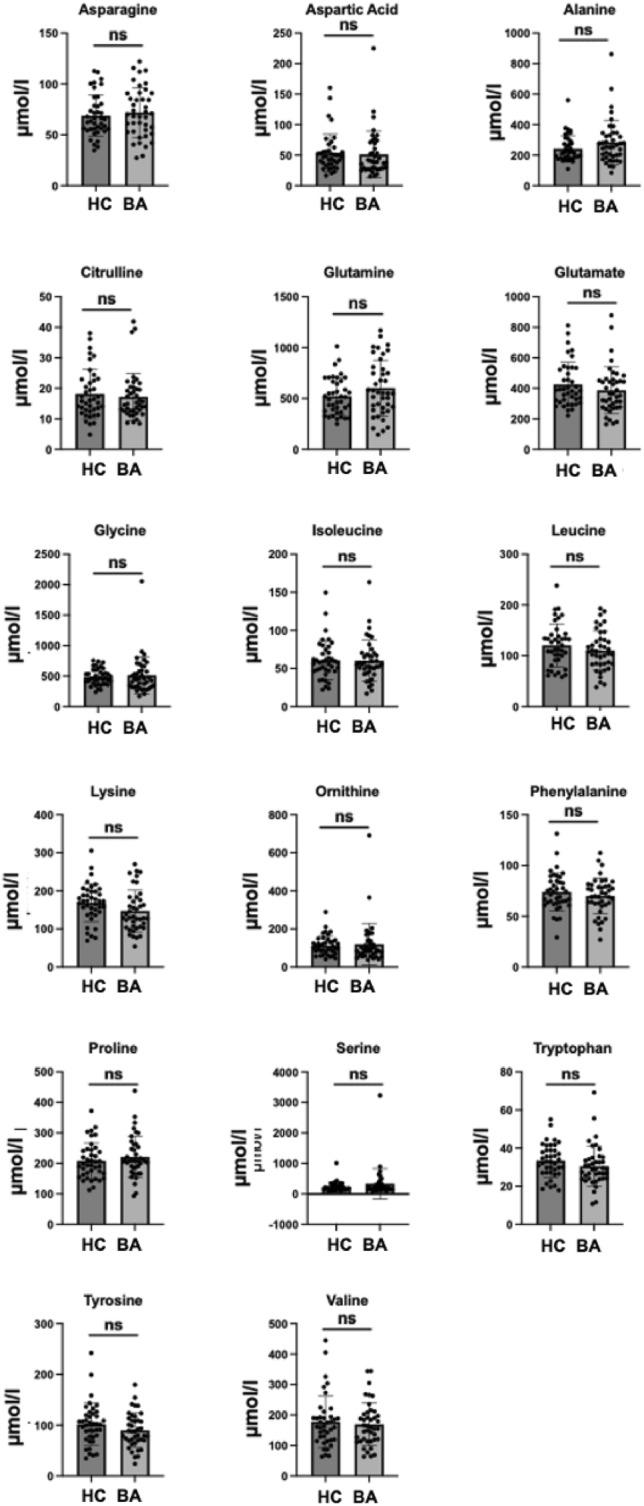
Other analyzed amino acids did not show significant differences between BA patients (*n* = 41) and healthy newborns (*n* = 40) at time of NBS (unpaired *t*-test; ns = not significant) (HC: healthy controls, BA: Biliary Atresia-Newborn Screening)

Regarding the remaining amino acids no significant differences were found between BA infants and healthy newborns at time of birth (Fig. 4) or for BA infants between time of birth and KPE (data not shown). No significant difference in Fisher index (ratio of branched-chain AAs (leucine, valine, isoleucine) to aromatics (phenylalanine, tyrosine)) was found between the two groups (data not shown).

### No correlation between amino acid profiles in newborn BA patients with clinical data and outcome

No significant differences were found between amino acid profiles of BA patients with syndromal form, cystic form or regular form of BA (Supplemental Fig. 1).

Pearson’s correlation coefficient was calculated for amino acid profiles and clinical parameters Bilirubin at Kasai, age at Kasai procedure, and ISHAK score. Spearman’s correlation coefficient was used for clinical outcomes during the recorded follow-up period (jaundice-free native liver survival, jaundice native liver survival, liver transplantation, death, no follow-up). No significant correlations between clinical parameters or outcome and amino acid levels were found (Supplemental Tables 1 and 2).

## Discussion

This study demonstrates that infants with BA exhibit a distinct amino acid profile during the immediate newborn period with higher levels of Methionine, Histidine, Arginine and Threonine compared to healthy neonates.

Of the elevated amino acids found in our cohort, methionine is the most extensively studied in regards to liver function and diseases. Hypermethionemia is regularly found in patients with liver cirrhosis with normalization of plasma levels after liver transplantation (Tietge et al., 2003). Methionine is essential for reducing oxidative stress in the liver by serving as a substrate for glutathione synthesis (Dever & Elfarra, 2010). Oxidative stress is suspected to play a crucial role in progressive cholestatic liver injury in BA infants (Tiao et al., 2007). BA infants show a significant decrease in antioxidant genes, including glutathione S-transferase alpha 1 (GSTA 1) which is negatively correlated with BA incidence and cirrhosis (Wang et al., 2019). Furthermore, glutathione levels as well as the activity of glutathione peroxidase, show an increase in BA patients after liver transplantation, suggesting an impaired glutathione metabolism in the native liver (Chen et al., 2015). In animal models of BA the toxin biliatresone induces bile duct injury resulting in altered cholangiocyte polarity and permeability as well as a loss of cell–cell adhesion (Waisbourd-Zinman et al., 2016). It also causes transient and selective depletion of intrahepatic glutathione, which appears to play an important role in determining susceptibility to this toxin-induced injury through its redox potential (X. Zhao et al., 2016). Differences in redox state between intra—and extrahepatic bile ducts have been detected and may explain why the extrahepatic biliary system is primarily affected in BA (X. Zhao et al., 2016). The increased methionine levels in our cohort may hence be due to increased oxidative stress in the liver already present at birth.

Interestingly, for both Arginine and Histidine, hepatoprotective effects have been described in animal models of liver disease (El-Batch et al., 2011; Mong et al., 2011; Rishi et al., 2011; Valero et al., 2000; Yan et al., 2009), but human data are scarce. As Histidine appeared to be the least stable metabolite when stored at room temperature (Fig. 1), the significant differences we detected in the NBS between BA infants and healthy newborns for Histidine are to be interpreted with caution. Studies on threonine in regard to human liver disease are equally limited, but serine/threonine kinases have been shown to be the most active in the setting of liver fibrosis (Creeden et al., 2022). Since Arginine, Methionine, and Threonine are part of the citric acid cycle, their elevation may speak of the reduced ability of the liver in BA infants, but the exact pathophysiological pathways remain to be understood.

Several studies have examined amino acid profiles in BA patients of older age than our cohort. A study in Chinese infants by Zhao et al. identified significant differences in multiple metabolites between BA (mean age 39.8 days ± 26.9 days) and the control groups, including the amino acids Glutamine, Glutamate and Tryptophan, none of which showed significant differences in our cohort (D. Zhao et al., 2014). Abukawa et al. tested plasma levels of amino acids in a small group of newborns aged from 24 to 61 days with neonatal intrahepatic cholestasis (Abukawa et al., 2001). The authors used BA patients as a control group, who showed elevated levels of Tyrosine, Threonine, and Methionine compared to healthy controls, with the latter two amino acids concurring with our findings. However, their second cholestatic control group of idiopathic neonatal hepatitis showed a similar amino acid profile, suggesting that our findings may not be specific to BA. Altered amino acid profiles have also been found in much older children (mean age 1.5 years) with BA with a total reduction of amino acids by 19% compared to healthy controls and significant differences between cohorts for molar fractional plasma amino acid profiles in a total of nine amino acids, among them threonine and methionine, but not arginine or histidine (Byrd et al., 1993).

Even tough there are discrepancies between the above studies and our results, the existing data confirms our finding of an altered amino acid metabolism in children with BA. Age-dependent changes of amino acid levels may explain the differences in amino acid profiles between the study cohorts but the exact pathophysiological mechanisms remain to be understood.

BA infants have been shown to have elevated serum levels of direct bilirubin within the first few days of life (Harpavat et al., 2011), almost certainly due to an impaired bile flow already present at this point. As the liver is an essential regulator of amino acid metabolism, shifts in amino acid profiles generally indicate impaired liver function (Paulusma et al., 2022). The altered amino acid profiles we found suggest that in addition to or because of the obstructed bile flow, liver function is also affected very early on. Our findings thus support the theory of a prenatal onset of BA as opposed to a postnatal trigger initiating the disease.

This underlines both the need and the possibility for a reliable newborn screening for BA. Due to its complexity, cost, and unclear specificity, the analysis of amino acid profiles as part of the regular NBS in order to detect BA cannot serve as a sufficient screening option at this point. Possible options to explore are measurements of Bilirubin levels (Harpavat et al., 2011) or BA specific markers, e.g. MMP7 (Yang et al., 2018), in dried blood spots, or combining a number of factors for optimal detection of patients. The search for a more practical and reliable alternative should be intensified in order to improve the outcome of BA through early diagnosis and treatment.

Due to its retrospective nature, our study has some limitations. The storage of DBS cards and their exposure to environmental factors retrospectively cannot be reliably determined and might pose a potential disruptive factor to our measurements. A cholestatic control group at the time of birth as well as a healthy control group at the time of Kasai would have facilitated better understanding and differentiation of our results. However, our data provide an interesting starting point for further research into the time of disease onset and amino acid metabolism in BA.

In conclusion, our NBS–DBS analysis showed that BA infants exhibit distinct amino acid profiles within 72 h after birth compared to healthy newborns as an early sign of impaired liver function, supporting the theory of a prenatal onset of the disease. This finding underlines the need for an appropriate screening parameter in order to improve the clinical outcome of patients through early diagnosis and treatment. Investigating the underlying mechanisms in amino acid metabolism may improve the understanding of pathophysiological processes in BA.

## Supplementary Information

Below is the link to the electronic supplementary material.

Supplementary file1 (DOCX 267 KB)

Supplementary file2 (DOCX 18 KB)

Supplementary file3 (DOCX 15 KB)

## Data Availability

No datasets were generated or analysed during the current study.

## Declarations

### Human or animal participants

This article does not contain any studies with animal participants performed by any of the authors. All procedures performed in the study involving human participants were in accordance with the ethical standards of the institutional and/or national research committee and with the 1964 Helsinki declaration and its later amendments or comparable ethical standards.
