# Supplementary material for: Infants with biliary atresia exhibit an altered amino acid profile in their newborn screening
Source: Metabolomics. 2024 Oct 5;20(5):109. doi: 10.1007/s11306-024-02175-2 (PMC11455667; doi:10.1007/s11306-024-02175-2)
Supplement: Supplementary file 1 — Supplementary file1 (DOCX 267 KB) [file 11306_2024_2175_MOESM1_ESM.docx]

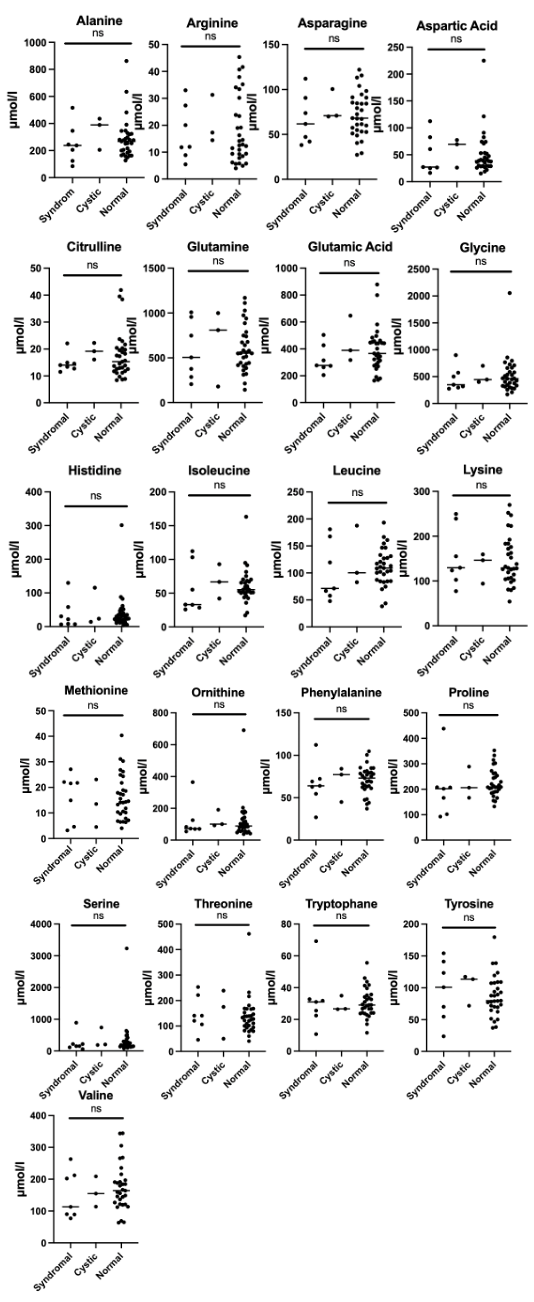


**Suppl. Fig.1:** No significant differences between amino acid levels was found between different clinical groups of BA patients: syndromal form (n=7), cystic form (n=3) and normal BA (n=31). (ANOVA, ns = not significant).
