# Supplementary material for: Infants with biliary atresia exhibit an altered amino acid profile in their newborn screening
Source: Metabolomics. 2024 Oct 5;20(5):109. doi: 10.1007/s11306-024-02175-2 (PMC11455667; doi:10.1007/s11306-024-02175-2)
Supplement: Supplementary file 2 — Supplementary file2 (DOCX 18 KB) [file 11306_2024_2175_MOESM2_ESM.docx]

|  | **Bilirubin at Kasai** | |  | **ISHAK Score** |  | **Time to Kasai** |  |
| --- | --- | --- | --- | --- | --- | --- | --- |
|  | **Pearson r** | | **p-value** | **Pearson r** | **p-value** | **Pearson r** | **p-value** |
| **Met** | -0.181 | | 0.26 | -0.231 | 0.15 | -0.289 | 0.07 |
| **His** | -0.163 | | 0.31 | -0.307 | 0.05 | -0.227 | 0.15 |
| **Arg** | -0.095 |  | 0.55 | -0.04 | 0.80 | 0.053 | 0.74 |
| **Thr** | 0.112 | | 0.49 | -0.176 | 0.27 | 0.098 | 0.54 |
| **Ser** | -0.124 | | 0.44 | -0.252 | 0.12 | -0.105 | 0.51 |
| **Ala** | -0.063 | | 0.7 | -0.037 | 0.82 | -0.029 | 0.86 |
| **Gln** | 0.091 | | 0.57 | -0.224 | 0.16 | -0.451 | 0.06 |
| **Pro** | -0.159 | | 0.32 | -0.086 | 0.6 | -0.171 | 0.28 |
| **Asn** | 0.149 | | 0.35 | -0.224 | 0.16 | -0.197 | 0.22 |
| **Gly** | -0.092 | | 0.57 | -0.367 | 0.21 | -0.171 | 0.29 |
| **Orn** | -0.106 | | 0.51 | -0.24 | 0.14 | -0.100 | 0.53 |
| **Ile** | -0.130 | | 0.42 | 0.132 | 0.42 | 0.142 | 0.38 |
| **Asp** | -0.001 | | 0.1 | -0.031 | 0.85 | 0.157 | 0.33 |
| **Val** | -0.125 | | 0.44 | 0.025 | 0.88 | -0.121 | 0.45 |
| **Cit** | -0.038 | | 0.82 | 0.115 | 0.48 | 0.015 | 0.93 |
| **Phe** | -0.279 | | 0.08 | -0.264 | 0.1 | -0.218 | 0.17 |
| **Leu** | -0.183 | | 0.25 | 0.111 | 0.5 | 0.128 | 0.43 |
| **Glu** | -0.196 | | 0.22 | -0.033 | 0.84 | -0.039 | 0.81 |
| **Trp** | -0.361 | | 0.06 | -0.279 | 0.08 | -0.301 | 0.06 |
| **Tyr** | -0.18 | | 0.26 | -0.053 | 0.74 | 0.043 | 0.79 |
| **Lys** | -0.171 | | 0.28 | -0.140 | 0.38 | -0.101 | 0.53 |

**Suppl. Table 1**: Calculation of Pearson Correlation for clinical data and amino acid levels without any significant correlation (n=41).
