# Supplementary material for: Infants with biliary atresia exhibit an altered amino acid profile in their newborn screening
Source: Metabolomics. 2024 Oct 5;20(5):109. doi: 10.1007/s11306-024-02175-2 (PMC11455667; doi:10.1007/s11306-024-02175-2)
Supplement: Supplementary file 3 — Supplementary file3 (DOCX 15 KB) [file 11306_2024_2175_MOESM3_ESM.docx]

|  | **Clinical Outcome** |  |
| --- | --- | --- |
|  | **Spearman r** | **p-value** |
| **Met** | 0.090 | 0.61 |
| **His** | 0.027 | 0.88 |
| **Arg** | -0.209 | 0.23 |
| **Thr** | 0.023 | 0.90 |
| **Ser** | -0.162 | 0.35 |
| **Ala** | -0.117 | 0.50 |
| **Gln** | 0.104 | 0.55 |
| **Pro** | -0.144 | 0.41 |
| **Asn** | 0.047 | 0.79 |
| **Gly** | -0.081 | 0.64 |
| **Orn** | -0.217 | 0.21 |
| **Ile** | -0.261 | 0.13 |
| **Asp** | -0.098 | 0.57 |
| **Val** | -0.051 | 0.77 |
| **Cit** | -0.075 | 0.67 |
| **Phe** | -0.097 | 0.58 |
| **Leu** | -0.234 | 0.18 |
| **Glu** | -0.068 | 0.69 |
| **Trp** | 0.005 | 0.98 |
| **Tyr** | -0.033 | 0.85 |
| **Lys** | -0.148 | 0.39 |

**Suppl. Table 2:** Calculation of Spearman Correlation for clinical outcome of patients (n=35) grouped into four categories: jaundice-free native liver survival (Bilirubin <20 μmol/l), jaundice native liver survival (Bilirubin > 20 μmol/l), liver transplantation, and death.
